# Supplementary material for: Active site specificity profiling datasets of matrix metalloproteinases (MMPs) 1, 2, 3, 7, 8, 9, 12, 13 and 14
Source: Data Brief. 2016 Feb 22;7:299–310. doi: 10.1016/j.dib.2016.02.036 (PMC4777984; doi:10.1016/j.dib.2016.02.036)
Supplement: Supplementary file 10 — Supplementary material [file mmc10.zip › WebPICS_hMMP7_T_1%/subsite_coop.html]

 

PICS results


- # About
- # Manual
- # Analysis
- # Results

- Redraw
- Seqlogo
- Dependency
- Coop

**Potential subsite cooperativity analysis for hMMP7\_T\_1%**   
Minimum difference for subsite dependency was set to +- 10 percentage-points  
Subsite dependency was checked for all positional occurences above 1 x natural abundance  
**Subsite dependency check is restricted to P3 - P3'**

|  |  |  |  |
| --- | --- | --- | --- |
| Fixed residue | Affected residue(s) | Change (percentage-points) | Vice-Versa change |
| P3\_A | P1\_S | 11.8 | 17.2 |
| P3\_E | P2\_Y | 11.6 | 23.1 |
| P3\_I | P3prime\_A | 14.2 | 10.5 |
| P2\_A | P1\_E | 11.6 | 11.6 |
| P2\_C | P3prime\_N | 52.6 | 28.4 |
| P2\_D | P1\_F | 10.5 | 16.4 |
| P2\_E | P1\_D | 13.0 | 15.9 |
| P2\_E | P1prime\_M | 14.3 | 35.2 |
| P2\_E | P2prime\_I | 10.6 | 10.2 |
| P2\_G | P1\_G | 19.5 | 25.0 |
| P2\_G | P3prime\_G | 12.6 | 11.2 |
| P2\_M | P2prime\_M | 34.5 | 21.6 |
| P2\_M | P3prime\_N | 25.5 | 19.7 |
| P2\_N | P3prime\_I | 10.9 | 13.2 |
| P2\_Q | P2prime\_H | 11.9 | 26.4 |
| P2\_Q | P3prime\_D | 12.4 | 11.3 |
| P2\_Q | P3prime\_Q | 13.1 | 13.1 |
| P2\_Y | P1\_N | 38.7 | 11.8 |
| P2\_Y | P3prime\_F | 15.2 | 10.9 |
| P1\_E | P3prime\_Q | 14.7 | 27.3 |
| P1\_F | P3prime\_H | 14.7 | 19.5 |
| P1\_H | P3prime\_N | 15.5 | 12.0 |
| P1\_P | P3prime\_D | 12.4 | 14.1 |
| P1\_Q | P2prime\_T | 12.0 | 14.6 |
| P1\_S | P1prime\_M | 12.2 | 22.6 |
| P1\_S | P3prime\_G | 16.8 | 11.2 |
| P2prime\_M | P3prime\_N | 14.3 | 17.6 |

  
**PICS analysis of protease: hMMP7\_T\_1%**  
291 cleavage sites analyzed  
PICS library made with (T)rypsin, (G)luC or (C)hymotrypsin:   
Cutoff for graphic display: 2 x natural abundance  

|  |  |
| --- | --- |
| Positional occurences  (table for total and relative (in %) values) | Occurences relative to natural abundance (table) |
|  |  |
